# Experiences of women seeking post-abortion care services in a Regional Hospital in Ghana

**DOI:** 10.1371/journal.pone.0248478

**Published:** 2021-04-20

**Authors:** Kenneth Setorwu Adde, Eugene Kofuor Maafo Darteh, Akwasi Kumi-Kyereme

**Affiliations:** Department of Population and Health, College of Humanities and Legal Studies, University of Cape Coast, Cape Coast, Ghana; Graduate School of Public Health and Health Policy, City University of New York, UNITED STATES

## Abstract

**Introduction:**

Abortions remain one of the highest contributors to maternal deaths in Ghana. In 2003, a policy on post-abortion care was introduced to help reduce abortion-related mortality and morbidity. However, depending on the method of pregnancy termination; women encounter varying experiences. This study examines the experiences of women seeking post-abortion care services in a Regional Hospital in Ghana.

**Materials and methods:**

In-depth interview technique was used to collect data from 20 purposively selected post-abortion care clients at the Volta Regional Hospital. Data were analysed manually using a qualitative content analysis technique.

**Results:**

The study found that medical abortion was the main method of pregnancy termination used by women who participated in the study to induce abortion. Spontaneous abortion, however, was attributed mainly to engaging in activities that required the use of excessive energy and travelling on bad roads by pregnant women. The study also revealed that, women do not seek early post-abortion care services due to stigma and poverty.

**Conclusions:**

We found that severity of pain from complications, stigma and financial constraints were factors that influenced women’s decision to seek post-abortion care services. Our findings also suggest that women who experienced spontaneous abortion mainly received financial and emotional support from partners and other family members. To encourage women to seek early post-abortion care services, the Ministry of Health and the Ghana Health Service should take pragmatic steps to educate women on the dangers associated with delay in seeking post-abortion care services and the factors that expose women to spontaneous abortions.

## Introduction

Globally, about 830 women die from pregnancy and childbirth-related complications every day, and abortion accounts for 8 percent of these maternal mortality cases [1]. Abortion is one of the global public health concerns, particularly affecting low- and middle-income countries where there are controversial perspectives as well as varied regulatory regimes [2]. While the developed world has witnessed a significant decline in abortion since the 1990s, same cannot be said for the developing world [3].

In Ghana, abortion is one of the main contributors to the maternal mortality rate [4]. More than one in every ten maternal deaths is attributed to unsafe abortion [5]. The high rate of maternal mortality that results from unsafe abortions can be attributed to social barriers such as provider attitudes [6], conscientious objection, and high stigma [7] attached to abortion in Ghana. These barriers make it difficult to effect appropriate policy reforms and changes that are necessary to address the problems posed by unsafe abortion [7]. Hence, women face many challenges in seeking abortion care [8]. Depending on the frequency, method, and location for seeking an abortion, women tend to have vast experiences motivated by diverse factors [9]. It is, however, worth mentioning that abortion complications do not only result from unsafe abortions, as both safe and unsafe abortions contribute to abortion complications [9].

Studies have indicated that most induced abortions result from unintended pregnancies which could be mistimed pregnancies, unplanned pregnancies or possibly unwanted pregnancies—all these have their associated experiences (see Warriner & Shah [10] and Finer et al. [11]). A woman who seeks a clandestine abortion may try some traditional techniques of varying efficacies and harmfulness, and these clandestine abortions have their own experiences [12]. Some methods of unsafe abortion include drinking toxic fluids such as turpentine and bleach or other drinkable concoctions mixed with livestock manure such as cow dung [12]. Others are a forceful manipulation of the abdomen and insertion of sticks and other objects into the vagina, cervix, or uterus [13]. Many of these techniques pose threats to a woman’s health and, sometimes, even her life [13]. If these methods fail to cause a complete pregnancy termination, the woman may then go to a pharmacist, a nurse, or a doctor known to provide abortion services [13].

According to the 2017 Ghana Maternal Health Survey, 20 percent of women aged 15–49 have ever induced an abortion. However, the percentage of women who have had an induced abortion in the last 5 years preceding the 2007 and 2017 surveys remained relatively unchanged, with 5 percent and 7 percent respectively [14]. A study conducted in Southern Ghana reported 17 abortions per 1,000 women of reproductive age [15] while another found that about 23 percent of women have ever had an induced abortion [16].

Although abortion is a criminal offence in Ghana, it is permitted when the pregnancy is a result of rape or incest, when there is a need to protect the mental or physical health of the mother, and when there is a malformation of the fetus, thus, making the abortion law in Ghana relatively liberal compared to other African countries [7]. Nonetheless, more than two-thirds of women who sought abortion turned to untrained providers or induced an abortion on their own [17, 18]. Concerning common abortion practices, findings from other studies suggest medical abortion through the use of Mifepristone and Misoprostol in Ghana [17, 19, 20]. Data from the Ghana Maternal Health Survey [14] showed 38 percent of the women who induced abortions indicated they used medical abortion, 27 percent used non-medical methods, and 24 percent used dilation and curettage (D&C) or dilation and evacuation (D&E) while 10 percent used other medical methods. Less common methods include: inserting an object, herbs, or other substances into the vagina; and drinking herbal concoctions [17, 20]. The latter methods of abortion are associated with several complications, thus requiring some post-abortion care (PAC).

To help deal with abortion complications and its effects on maternal mortality and morbidity rates, a policy on post-abortion care was introduced in Ghana in 2003 [21]. In 2006, the Ghana Health Service and the Ministry of Health also published protocols for the provision of safe abortion services to help reduce the barriers to safe abortion. This was to help reduce abortion-related mortality and morbidity [22]. However, abortions remain one of the highest contributors to maternal deaths [4]. Though Ghana has made efforts to increase access to post-abortion care, the quality of service is poor, underfunded and has low visibility [23]. It is, therefore, important to promote an equitable and reasonable healthcare seeking environment to improve reproductive and maternal health in Ghana.

Various issues concerning abortion have been studied in Ghana. For instance, studies have been conducted on the practice of abortion in Ghana and attitudes towards abortion (see Morhe et al. [24]; Sundaram, et al. [25]; GSS [20]). Others have focused on post-abortion experiences in Ghana (see Esia-Donkoh et al., [26]; Rominski et al., [27]). Esia-Donkoh et al., [26] targeted women who received safe abortion services at a health facility while Rominski et al., [27] focused on post-abortion contraception choices of women. This notwithstanding, there appears to be a paucity in the literature on the health-seeking behavior of women who sought post-abortion care. The current study, therefore, explores the experiences of women who received post-abortion care services in the Volta Regional Hospital. This will enable us to have a clear view of the health-seeking behavior of women who have had unsafe and spontaneous abortions and suggest feasible measures to improve post-abortion care services and maternal health care.

## Materials and methods

The study relied on the interpretivist paradigm on the backdrop that it provides a better way of understanding real-life experiences of post-abortion care clients concerning pregnancy termination. The data for the study was collected from post-abortion care clients at a regional level health facility in Ghana. A regional level health facility in Ghana serves as a secondary referral point for all health facilities in a region. The Volta Regional Hospital is one of the state-owned regional referral hospitals. Obstetrics and gynaecology care are some of the critical services available at the facility. The 2017 Ghana Maternal Health Survey indicates that the Volta Region recorded one of the highest incidences of induced abortions and miscarriages. About 16 percent of women in their reproductive age had ever had an abortion.

Clients who were at the facility to seek post-abortion care services were purposefully selected with the help of health professionals. At a point where the PAC clients were about to be discharged, the service providers informed the PAC clients of the study. Clients who were willing to partake in the study were approved by the service provider for their psychological and physical readiness for an interview before they were included. The interview process was carried out by the lead author who had had two years of training and experience in conducting sensitive interviews before this study. A male interviewer was used against the backdrop that Ghanaian women reported more prior sexual activity to male interviewers [28]. Women who were willing to partake in the study were provided with consent forms to sign/thumbprint. They were then allowed to select their place and time of convenience for the interview to be carried out. A psychologist was on standby to be called upon in instances of re-traumatization during the interview process. The interviews were recorded and transcribed. On the average, an interview lasted for about 45 minutes. Informed by data saturation, a total of 20 women, made up of 8 clients who induced abortions and 12 clients who had spontaneous abortions participated in the study. Guest, Bunce, and Johnson [29] theorize that a sample size of six interviews could be enough for the development of meaningful themes and useful interpretations.

The in-depth interviews were conducted with the aid of an interview guide. The guide had two sections (A and B). Section A focused on the background characteristics of the participants, and section B focused on the experiences with pregnancy termination and the support received from family and friends. Respondents were asked to describe their pregnancy termination experiences. Among the key issues were what informed their decisions to seek post-abortion care and the form of support they received while seeking post-abortion care. Ethical clearance for the study was obtained from the Ghana Health Service Ethical Review Committee (GHS-ERC: 14/10/15). Permission to conduct the study was also obtained from the management of the hospital.

Data were analysed manually using a qualitative content analysis technique. A qualitatively-oriented text analysis based on the principles of the consolidated criteria for reporting qualitative research (COREQ) was adopted. To ensure that the findings of the study were credible and unbiased, the recorded interviews were transcribed by an experienced researcher. The transcripts were cross-checked by a member of the research team. The data were coded independently by an expert qualitative data coder and the lead author. To ensure intercoder concordance, the authors and the expert qualitative data coder met afterwards and reviewed the coding outcomes. Two experts in qualitative research cross-checked the codes for consistency. Following the jointly reviewed codes, themes and emerging issues were developed to mirror the narratives. Finally, quotes from the study participants were used to corroborate the issues discussed.

## Results

### Socio-demographic characteristics of participants

Table 1 presents the socio-demographic characteristics of the participants. Sixteen respondents were in their 20s and three were aged 30–39 years. Those who had attained basic education were seven and six had tertiary education. Nineteen of the participants were Christians and one was a Muslim. Eleven of the participants were never married and nine were married at the time of the study. The participants were mainly dressmakers (5), civil servants (5), and traders (4).

**Table 1:** Socio-demographic characteristics of participants.

Variable	Frequency (n = 20)	Percentage (%)
**Age**		
< 20	1	5.0
20–29	16	80.0
30–39	3	15.0
**Level of education**		
No education	2	10.0
Primary	3	15.0
JHS	7	35.0
SHS	2	10.0
Tertiary	6	30.0
**Religion**		
Christian	19	95.0
Muslim	1	5.0
**Marital status**		
Never married	11	55.0
Married	9	45.0
**Occupation**		
Dressmaker	5	25.0
Trader	4	20.0
Farmer	2	10.0
Civil servant	5	25.0
Student	2	10.0
Hairdressing	2	10.0
Total	20	100.0

Source: Field Data, 2016.

### Abortion experiences of respondents

Abortion experiences among women include the decisions they had to make, their emotions, physical experiences, strategies they used, including health care advice and dealing with clandestine medical abortions [25]. The main experiences which emerged from the study included: abortion strategies, perceived causes of spontaneous abortion, decisions to seek post-abortion care, and support received from family and friends during the abortion processes.

#### Abortion strategies

Medical abortion–Mifepristone and Misoprostol—is widely used by women in an attempt to terminate pregnancy [19]. The results revealed that most of the respondents induced abortion at home, using the medical abortion method which involved swallowing of pills. According to some of the respondents, they had access to pills from other people, especially friends. A client who had used a pill to terminate a pregnancy shared her experience as follows:

*“I took some medicine*. *I was discussing the pregnancy with a friend and she told me she knows some medicine*, *so she will bring it to me*. *She brought me the medicine and I used it.”*

–Seamstress, 20–29 years.

Adding to this, some of the respondents themselves were well aware of the pills they had to take to terminate the pregnancy. A client who got the pills herself had this to say:

*“I just bought some medicine and took it*. *The medicine is called Cytotec*. *After taking it*, *I stayed indoors for a while I think for about 8 hours and I started feeling cramps and not long after that the blood started flowing.”*

*–*Caterer, 20–29 years.

These experiences indicate that some women who induce abortion resort to medical abortion and may seek information regarding the pills to be taken from friends. A plausible explanation is that women may not want people to know about their abortion. As such, women would rather prefer to have a clandestine abortion. This could be as a result of women’s attempts to avoid the stigma attached to abortions at the societal level.

#### Perceived causes of spontaneous abortions

The study found that not all women deliberately induced abortion. The data revealed that 12 out of the 20 respondents had their pregnancies terminated unintentionally. Among the respondents who had their pregnancy terminations classified as “spontaneous abortions,” two main sources were observed: first, those whose pregnancies were aborted as a result of a second party, and second, those who lost their pregnancies unintentionally through their actions. The following extracts demonstrate the experiences of respondents who experienced spontaneous abortions as a result of a second party.

“We were given a dewormer at… after we took the medicine … our headmaster told us that the medicine aborts pregnancy.”

*–*Student, <20 years.

Another closely related view to the above was expressed by a woman who indicated that her abortion was as a result of physical abuse by her husband. She had this to say:

*“For this abortion*, *I will say it is my husband that gave me problems that resulted in the abortion*… *We were struggling with each other and someone else came to hit me from behind on my waist*. *It was his girlfriend who did that.”*

*–*Seamstress, 20–29 years.

Some other respondents had their pregnancies terminated by their actions, though unintentional. Some of the respondents expressed experiencing spontaneous abortions as a result of their engagement in activities that demand the use of excessive energy, such as cooking some meals and farming activities. A respondent had this to say:

“*Hmmm*, *I don’t know how to say it*. *I went to the farm to do some weeding*. *I was back from the farm and I realized I was bleeding so I came to the hospital and as soon as I got here the fetus itself came out.”*

*–*Student, 20–29 years.

Another respondent indicated that she noticed there was something wrong with her pregnancy while preparing “banku” (Ghanaian dish). Although the preparation of this meal requires the use of excessive energy, it is likely the abortion resulted from other health issues. The respondent had this to say:

*“…I was preparing ‘banku’ when the blood poured out of me*. *I couldn’t even continue cooking the ‘banku’*. *I had to leave it on the fire…”*

–Hairdresser, 20-29years.

Some other respondents also experienced spontaneous abortions as a result of travelling on bad roads. To such respondents, their pregnancies got terminated because of the bumpy nature of the roads in their communities. The major means of travelling used by these respondents was a motorbike. One respondent surmised:

*“… I can’t exactly tell what happened but I did some rounds travelling around to my dad’s place and other places on some very bad roads with a motorbike so when I got back*, *I started bleeding…”*

*–*Fashion designer, 20–29 years.

To buttress this quotation, another respondent had this to say:

“…I took a ride on a motorbike and it bumped into a pothole and I started feeling pains in my abdomen only to come to the hospital and be told I have lost the pregnancy.”

–Trader, 20–29 years.

From the above, it is evident that the women associate the loss of their pregnancies with the activities they were engaged in before the pregnancy loss. These activities demand the use of excessive energy. Although these activities could be contributory factors to the pregnancies loss we also believe there might be some other medical conditions which could have been aggravated by these activities to result in pregnancy losses.

One other respondent indicated that her spontaneous abortion was as a result of an accident that occurred at home. She fell, and this caused some pregnancy complications which later resulted in the unintentional termination of the pregnancy. She said this:

*“I slipped on the floor*. *Where we were staying at first had a cemented floor so when it rained the floor was slippery and I slipped and fell*. *I started bleeding so I went to the hospital.”*

–Fashion designer, 20–29 years.

Notwithstanding that only one respondent said she lost her pregnancy as a result of an accident at home, the above experience shows that accidents at home could also result in spontaneous loss of a woman’s pregnancy.

#### Decisions to seek post-abortion care

The study observed that different situations informed women’s decisions to seek PAC services. For instance, women who induced their abortion decided to seek PAC only at the point where they felt the pain was becoming unbearable and needed professional help in dealing with their situations. A respondent had this to say:

*“My abdomen was aching severely and I went to the hospital*. *It started as some normal pains but was increasing so when I couldn’t bear it any longer*, *I went to the hospital.”*

–Student, <20 years.

One other respondent who also waited till the pain became severe and unbearable before seeking PAC services had this to say:

*“Well*! *When the thing* [abortion] *happened*, *I didn’t come to the hospital immediately*. *I stayed home for some time and I started feeling pains in my waist*. *I went to urinate and saw blood in my underwear*. *When the pain was becoming too much*, *I pushed*. *After pushing*, *the blood started coming out of me like water*. *I almost died out of that*. *The blood was just coming out like water for a long time so I became weak”*

*–*Seamstress/trader, 20–29 years.

The findings of the study suggest that some of the women who experienced complications from induced abortion were scared of stigma by society. This, to a large extent, affects the decision-making of some women to seek help at healthcare facilities. Their position was that they would not want anyone to know they have had an induced abortion; hence, they preferred to hide their situation and suffer in silence. A respondent remarked:

*“The thing became unbearable for me before I decided to go to the hospital*. *I was feeling pains but I was feeling shy and didn’t want anybody to know what was happening to me so I refused to go to the hospital*. *But later on*, *I couldn’t breathe again so they had to hire a taxi to take me to the hospital.”*

*–*Seamstress, 20–29 years.

Although the women were in pain, they were reluctant to seek early treatment from a health facility and delayed until the pain was unbearable. This could be attributed to the fact that women feared being stigmatized for having induced an abortion. It could also be that these women were not aware of their right to comprehensive abortion care and, as such, were scared to seek early post-abortion care.

Women who experienced spontaneous abortion also delayed in seeking post-abortion care services at the hospital for various reasons. One of these reasons is the lack of or expiration of their national health insurance subscription. This prevented some women from seeking care at the hospital since they did not have money to pay for the services at the health facility. One woman had this to say:

*“…if not for the fact that my national health insurance card had expired*, *I would have come earlier and not wait for it to become this serious.”*

*–*Hairdresser, 20–29 years.

Nonetheless, multigravida women and women who experienced spontaneous abortion did not take long in seeking care at the hospitals. They were quick to report at the hospitals when they noticed a change or felt something was not in the right order. A multigravida respondent noted:

*“I know that during pregnancy you are not supposed to see blood*. *I have given birth before and I know the process so immediately I saw something come out of me I knew something was wrong and I had to go to the hospital.”*

–Fashion designer, 20–29 years.

To corroborate this, another multigravida respondent noted:

*“I was feeling cold and all of a sudden*, *I started feeling dizzy*. *I realized something was wrong with me so I was rushed to the hospital.”*

–Farmer, 30-39years.

The findings show that lack of or the expiration of national health insurance and the fear of stigma are factors that delay the health-seeking behavior of women seeking post-abortion care. However, multigravida women and women who experienced spontaneous abortion were quick to report for early post-abortion care. Seeking early post-abortion care by the multigravida women could be as a result of earlier experiences with pregnancies. The multigravida women and women who experienced spontaneous abortion could have been opportune to learning the importance of reporting for early treatment during antenatal care for their previous pregnancies. Knowing the dangers of delay in health-seeking, multigravida women would probably be more concerned with their health as compared to the stigma attached to abortion. This could be a probable reason why these multigravida women and women who experienced spontaneous abortion seek early PAC services.

#### Support received from family and friends

We observed that 16 of the women received support from their partners, family, and parents in seeking post-abortion care at the hospital. This support mainly came in financial and emotional forms for women who reported with spontaneous abortion while women who reported with induced abortion mainly received only financial support. In most cases, those who received support from their partners and families acknowledged that the support was very critical in their decisions to seek care as well as their general experiences with the procedure. For instance, a respondent who reported with spontaneous abortion had this to say:

*“Yes*, *my husband*. *He has been very helpful right from the beginning till now*. *He was there for me by going to the hospital with me and when I was admitted*, *he came to visit regularly and bought food for me as well as being supportive in comforting and consoling me to go through the process.”*

*–*Caterer, 20–29 years.

To corroborate the quote above, another respondent who received financial support for PAC from her family and partner had this to say:

*“Yes*, *my father was a watchman and he is now on pension but takes some monthly pay of about GHS800 so he went for it and the boy who impregnated me also added some as well as my elder brother and they took me to the hospital*. *They also visited me from time to time.”*

*–*Student, <20 years.

On the other hand, four of the respondents did not have any support from their immediate families and partners for PAC services. This resulted in being left stranded at the hospitals since they could not afford their bills. Consequently, they were kept at the hospitals against their will, although they had fully recovered from their conditions. This was what a respondent had to say:

*“No one helped me*. *Even as I am here right now*, *I have been discharged but because of my bills*, *I can’t go home*. *My sister was the one who got me a taxi but even that there is a man in our house who is a driver so he decided to help me by bringing me*. *Aside from that*, *I have not received any form of help from anybody again.”*

–Seamstress, 20–29 years.

To corroborate this, another respondent said this:

*“Please no*, *there is no one*, *even up till now I don’t have anybody who will pay my bills for me.”*

–Hairdresser, 20-29years.

The findings show that a larger proportion of the PAC clients received support from their family and friends. This could be ascribed to the family support system in many sub-Saharan African countries where family members tend to be each other’s keepers.

## Discussion

We explored the experiences of women seeking PAC services. The results showed that women who induced their abortions relied on medications to terminate their pregnancies. This could be as a result of the fact that medical abortion offers women the privacy to terminate their pregnancies without having to consult others in health facilities. This also helps to reduce stigma, as when done successfully, no one might even know of it. This is in line with a study conducted by Sedgh [17], which revealed that most women who had an abortion, induced abortion on their own. Other studies also observed that women generally preferred medical abortions to other pregnancy termination techniques [19, 20]. Given the high usage of medical abortion, it is important to strengthen public education for women and pharmacists on the right medication for medical abortion to reduce the risk of women using illegal methods of abortions. The findings are, therefore, suggestive of urgent need for the government of Ghana to acknowledge that safe abortion care should constitute a critical part of the interventions to reduce maternal morbidity and mortality.

The study also found that the spontaneous termination of pregnancies among women was mostly perceived as a result of engaging in strenuous physical activities. This is, however, not medically proven, as it is from the respondents’ lay point of view. This corroborates the findings of Wong et al. [30] that there is an elevated risk for women working in jobs that required the use of strenuous physical energy. It would, therefore, be imperative for women to know their health conditions before indulging in certain activities while pregnant.

It was observed that, whereas various factors influenced the time a woman decided to seek professional healthcare for post-abortion complications, the main factor was the severity of the pain resulting from the complications. Stigma and poverty, nonetheless, also played a vital role in influencing the decision-making process. The delay by women seeking PAC services could be attributed to the fact that women would like to suffer in silence to protect their privacy rather than suffer the stigma attached to abortion since in most developing countries, abortion is a religious, moral, and political issue instead of a medical or public health issue [3]. This corroborates the findings of Finer and Henshaw [31], who argued that most women allow a longer time to elapse between abortion and the time for seeking care. This could be attributed to the stigma associated with induced abortion in developing countries, coupled with the laws that render an abortion legal only under certain conditions [7]. Consequently, more advocacy has to be done to encourage women to seek early PAC services to reduce the rate of late reporting for PAC services when complications arise.

The study has revealed that women who presented with spontaneous abortion received both emotional and financial support from family and friends while their counterparts who presented with induced abortion mainly received only financial support. A possible explanation is that spontaneous abortion could result in post-traumatic disorder [32] hence the need for emotional support for this group of women. There is therefore the need for well-targeted and responsive post-abortion counselling services for PAC clients.

Despite the important findings made from this study, it is imperative to indicate its possible limitations. For instance, the study was delimited to clients from only one health facility and a small sample size which limited the study from being generalized to the general Ghanaian society. This, however, did not limit the validity and trustworthiness of the study, since the necessary steps were followed to make the instrument valid and trustworthy.

## Conclusions

The study established that women who induced their abortions preferred to do so through medical abortion. However, the women who participated in the study are of the view that their spontaneous abortions were mostly as a result of carrying out activities that required the use of excessive energy, and travelling on bumpy roads. The study also concluded that stigma served as a barrier that hindered women from seeking PAC services. Furthermore, the study has shown that women who experienced spontaneous abortion for PAC services mainly received financial and emotional support from their partners and other family members. To encourage women to seek early treatment, the Ministry of Health and the Ghana Health Service should take pragmatic steps to educate women on the dangers associated with delayed post-abortion care and the factors that expose women to spontaneous abortions.

## Supporting information

S1 File (DOCX)

S2 File (DOC)

S3 File (PDF)
