# Supplementary material for: Experiences of women seeking post-abortion care services in a Regional Hospital in Ghana
Source: PLoS One. 2021 Apr 20;16(4):e0248478. doi: 10.1371/journal.pone.0248478 (PMC8057598; doi:10.1371/journal.pone.0248478)
Supplement: S1 File — (DOCX) [file pone.0248478.s001.docx]

**University of Cape Coast**

**College of Humanities and Legal Studies**

**Faculty of Social Sciences**

**Department of Population and Health**

**In-Depth Interview Guide for Post Abortion Care (PAC) Clients**

**Background Information**

| Participants No. |  |
| --- | --- |
| Date of Interview |  |
| Name of interviewer |  |
| Age |  |
| Educational level |  |
| Religion |  |
| Occupation |  |
| Place of residence |  |
| Remarks/ context |  |

**Abortion experiences**

1. How many abortions have you ever done?
2. How was the last abortion done?
3. Can you please tell me about how easy the method you used was?
4. Can you please tell me the cost of the last method and who paid for it?

**Experiences before seeking post abortion care**

1. What made you decide to come for the service?

- When was the decision made to seek care?
- Who made the decision for you to present to the hospital?

1. Can you please tell me if you had any support from anybody to seek post abortion care?

-**Probe** for the type of support and from which people
