# Supplementary material for: Experiences of women seeking post-abortion care services in a Regional Hospital in Ghana
Source: PLoS One. 2021 Apr 20;16(4):e0248478. doi: 10.1371/journal.pone.0248478 (PMC8057598; doi:10.1371/journal.pone.0248478)
Supplement: S2 File — (DOC) [file pone.0248478.s002.doc]

RECORDING 1

A: What’s your age?

B: 23years

A: Level of education

B: Informal

A: Are you Christian?

B: Yes

A: Which church do you fellowship with?

B: Zion Temple

A: Are you married

B: No, I am not

A: What’s your occupation?

B: I am a seamstress

A: Your place of residence

B: Ho

A: Have you ever done an abortion

B: No, this is the first

A: How did you do it?

B: I used drug

A: Did you buy the drug yourself?

B: No, I was speaking with a friend and he said he knew a drug so he would get it for me

A: Was it easy?

B: No, it was very hard and painful

A: Can you tell me something about it?

B: When I used the drug, the baby wasn’t coming out but I was in pain until I started bleeding few days later. I used the medicine in December but nothing happened. We entered January before I was feeling pains

A: How much did you spend on the drugs?

B: I don’t know any abortion drug so my friend bought it for me, he didn’t collect any money from me. I sold some clothes for him but he didn’t tell me whether he bought the drug from the money he owed me.

A: Did the baby come out?

B: No, it came out only when I came to the hospital

A: Who treated you at the hospital?

B: Doctors, midwives and nurses. But at the time the foetus came out, there was a nurse on duty who cleaned me up. She gave me a chamber pot and asked me to sit on it. With her gloves on, she then used something which looked like scissors to cut the baby from the umbilical cord. She then inserted her fingers into my private part and brought out the rest of the things.

A: Have you received any counselling?

B: They wanted to perform evacuation of the uterus but because I don’t have money I refused.

A: Have you been taken through Family Planning Services?

B: No, but one of the nurses told me to consider using any family planning method

A: Why did you decide to come to the hospital?

B: To be frank, I didn’t wish to come the hospital but I realized if I wasted about an hour, I would die so my sisters brought me to the hospital. I was in pain and was feeling shy so I didn’t want anybody to know what was happening to me but later, I couldn’t breathe so my sisters chattered a taxi and brought me here.

A: Did you receive support from anyone?

B: I didn’t receive any support from anyone. I have been discharged but because I couldn’t pay the bills, I am still here. My sisters brought me. The driver stays in our area so; it could be that he offered to help.

A: Can you tell me about the attitude of the Service Providers?

B: The nurses are good and the doctors are caring. Nobody is maltreating me. They talk to me politely. They only prevent me from moving about because a lady had escaped from them and they think I will do same.

A: Are you satisfied with the services?

B: Yes, I am satisfied because we didn’t have money when we came but they started attending to me before prescribing some drugs for us to buy.

A: What are some of the challenges you are facing?

B: No one had actually said anything bad to me. I am only facing financial challenges. I am not able to feed well because I don’t like the food. I am not able to take my bath because the tap isn’t flowing

A: Any further comments?

B: I don’t have anybody to help me. I am appealing to them to allow me to sign an undertaking and grant me some time to go and look for money to pay for the bills. They can take me to know to my house but they are not making any effort.

RECORDING 2

A: What’s your age?

B: 22years

A: Level of education

B: JHS

A: Are you Christian?

B: Yes

A: Are you married

B: Yes, I am

A: What’s your occupation?

B: Hairdressing

A: Your place of residence

B: Kpetoe

A: Have you ever done an abortion

B: No, I have a child, this is the second pregnancy but I have miscarriage.

A: Does it mean you did not take any abortion drug?

B: No, I travelled on motorbike from a far distance to my husband and I started to bleed so I was brought here.

A: Can you tell me something about it?

B: When I used the drug, the baby wasn’t coming out but I was in pain until I started bleeding few days later. I used the medicine in December but nothing happened. We entered January before I was feeling pains

A: Who attended to you at the hospital?

B: Doctor

A: What happened and what kind of treatment did you receive?

B: The whole thing started at night so I was sent to a clinic where I was infused five times and hence transferred here. I was infused here too. I was taken through the evacuation of the uterus and some vaginal examinations.

A: Did you feel any pain?

B: Yes, I felt pain but I was injected to reduce the pain.

A: Have you received any counselling?

B: No.

A: Have you been taken through Family Planning Services?

B: I had done one but no test was conducted to find out if I was pregnant so I still have it.

A: Have you had any Family Planning Services here?

B: No

A: Have they spoken about it to you?

B: Yes. They told me when I am ready to take another one, I should come for first one to be removed because it is no longer effective.

A: Have you been educated about the various Family Planning options?

B: Yes, I am told there are different types and how they work differently. They have for 5years, 3 years, 1 year and the monthly ones

A: Why did you decide to come to the hospital?

B: I fainted when the whole thing started so I only realized I was brought here after I had recovered.

A: Did you receive support from anyone?

B: No please I have no one to pay my bills. My husband came but he has no one to help him pay the bills.

A: Can you tell me about the attitude of the Service Providers?

B: They are very caring. They take care of everyone.

A: Are you satisfied with the services?

B: Yes, I am. Sometimes, people die in such cases but by the grace of God, they attended to me properly and I am alive.

A: What are some of your challenges?

B: Financial challenges already existed. Distance wasn’t a challenge. We charged GH₵60.00 for the transportation.

A: Any further comments?

B: We shall be happy if any philanthropist could come to our aid to our aid to help us pay our bills so that we can be discharged.

RECORDING 3

A: What’s your age?

B: 25years

A: Level of education

B: JHS

A: Which Religion?

B: Muslim

A: Are you married

B: Yes

A: What’s your occupation?

B: Unemployed

A: Your place of residence

B: Sokode Lokoe

A: Have you ever done an abortion

B: No

A: Did you abort this one yourself?

B: No, I had a miscarriage

A: How did it happen?

B: I woke up and realized I was bleeding so I came to the hospital

A: How were you treated when you came to the hospital?

B: They did vaginal examination and the evacuation of the uterus.

A: Who treated you at the hospital?

B: Doctor

A: Were you taken through any counselling?

B: No.

A: Have you been taken through Family Planning Services?

B: No

A: Why did you decide to come to the hospital?

B: Because of the excessive bleeding

A: When did you decide to come to the hospital

B: Immediately I realized I was bleeding

A: Did you receive any help from anyone and in which form?

B: Yes, my Church member. She was at home so I went to call her and she brought me here. My husband also supported me financially.

A: Can you tell me about the attitude of the Health workers?

B: It’s fine. They talk to me politely.

A: Are you satisfied with the services?

B: Yes, I am. Their prompt response has made the pain to be minimized.

A: What are some of the challenges you are facing?

B: No, except that my house is far from the hospital. I were quick to attend to me. I was given the OPD card immediately I got there.

A: Is the hospital environment clean?

B: It is very clean

A: Are there enough beds?

B: Yes

A: Are visitors allowed to visit you?

B: Yes

A: Are you allowed to practice your religion?

B: Yes, all religions are allowed to fellowship; Muslims fellowship outside the ward because of the ablution but Christians do theirs inside

A: Why didn’t you go to any other health facility?

B: This is where I had my first delivery so I came back here

A: Why did you have your first delivery here?

B: I just like the care they give to everyone in terms of food and they sell to us on credit.

A: Any further comments?

B: Government should include other services like evacuation of the uterus and infusion in the health insurance so that those who cannot pay for those services can also be catered for.

RECORDING 5

A: What’s your age?

B: 28years

A: Level of education

B: Primary

A: Which Religion?

B: Christian

A: Are you married

B: No, but I had a boyfriend

A: What’s your occupation?

B: Hairdressing

A: Your place of residence

B: Agortime-Kpetoe

A: Have you ever aborted any pregnancy

B: I don’t abort pregnancies. I was pregnant with twins but the membrane surrounding the foetus got damaged. I delivered but the babies died 2 weeks and one month respectively. This is my second miscarriage.

A: How did it happen?

B: We went to fetch water and felt wet. I later felt I passed out something so my aun asked me to visit the toilet to check. When I got behind the toilet, I realized that the foetus had come out but the placenta. At about 7p.m, the placenta wasn’t coming out so I was rushed to a hospital in Togo. I was injected, infused and I felt weak and could not eat anything.i was told the evacuation equipment was not at the facility and if the ejection is not done, it would affect me in future so I returned to Kpetoe to seek treatment. I could not sleep for three nights. When I finally arrived in the hospital, I was driven in a wheelchair.

A: How were you treated when you came to the hospital?

B: An equipment was passed through my vagina. I was injected before the evacuation. It was painful.

A: Who treated you at the hospital?

B: a female doctor

A: Were you taken through any counselling?

B: No, not yet

A: Have you been taken through Family Planning Services?

B: No

A: When did you decide to come to the hospital

B: Because I was told that I would be affected if the evacuation of the uterus is done.

A: Did you receive any help from anyone and in which form?

B: My aunt sent me to the hospital but she asked me to refund all monies she spent. So I can say I don’t have anyone to help me

A: Can you tell me about the attitude of the Health workers?

B: I was badly treated the last time I visited the hospital but those who treated me currently are good.

A: Are you satisfied with the services?

B: Yes, I am. There have not been any disturbances from them.

A: What are some of the challenges you are facing?

B: My expired health insurance card hindered me from assessing early treatment. The distance from Kpetoe to this place is far. I also spent a long time at the OPD for a card because I had forgotten the old one at home.

A: Is the hospital environment clean?

B: They always clean the place but the unavailability of water now, one of the W.Cs is in a mess

A: Are there enough beds?

B: Yes

A: Are visitors allowed to visit you?

B: Yes but at designated times

A: Are you allowed to practice your religion?

B: They do not say anything tome about that but one can have solemn prayers. Sometimes, people come here to pray with us

A: Why didn’t you go to any other health facility?

B: The place is neat.

A: Why did you have your first delivery here?

B: I just like the care they give to everyone in terms of food and they sell to us on credit.

A: Any further comments?

B: I only brought GH₵300.00 to the hospital with the hope that the health insurance would cover all services. I had to pay GH₵130.00 for the evacuation.

A: I would like to know how the evacuation of the uterus was done

B: I only saw that some equipment was inserted in vagina to remove the clot

A: Was it only the clot?

B: The foetus was removed before the clot

A: Did you feel pains?

B: Yes

A: About how many minutes?

B: About 1 hour

RECORDING 6

A: What’s your age?

B: 29years

A: Level of Education

B: Tertiary

A: Which Religion?

B: Christianity

A: Are you married

B: Yes

A: What’s your occupation?

B: Catering

A: Your place of residence

B: Tema

A: Have you ever done an abortion

B: I had three miscarriages but I had one abortion.

A: How did you do the abortion?

B: I used drugs

A: Was it difficult?

B: No, it wasn’t. I inserted the drug in my private part and by morning, the foetus came out

A: How much did we spend?

B: I spent about GH₵50.00 but I am currently having miscarriages. The first was 2months and the second was 4months

A: Can you tell me what happened?

B: I got to the hospital about 11:00p.m. I was infused before the doctor was called to attend to me.

A: How were you treated when you came to the hospital?

B: The miscarriage actually happened at home. I bled profusely so when I came so they did evacuation of the uterus to remove the clot.

A: Who treated you at the hospital?

B: Doctor

A: Were you taken through any counselling?

B: No. A midwife only told me to wait until the wound heals before get pregnant again

A: Have you been taken through Family Planning Services?

B: No

A: Why did you decide to come to the hospital?

B: I went to hospital where I later realized that I had a miscarriage after 8 weeks and had to be flashed off. I requested from my husband to see our Pastor in Ho. We were with our Pastor when I felt pains and I was rushed to this hospital.

A: Did you receive any help from anyone and in which form?

B: Yes, my husband supports me financially.

A: Can you tell me about the attitude of the Health workers?

B: It’s fine. They are polite and caring.

A: Are you satisfied with the services?

B: Yes, I am. They make sure everyone takes her bath and they support those who cannot bath to do so.

A: What are some of the challenges you faced?

B: I had a heavy flow of blood before I got here.

A: Is the hospital environment clean?

B: It is very clean

A: Are there enough beds?

B: Yes

A: Are visitors allowed to visit you?

B: Yes

A: Are you allowed to practice your religion?

B: I haven’t actually experienced anything like that

A: Why didn’t you go to any other health facility?

B: I was rushed her by my husband

A: Any further comments?

B: I am happy for the services rendered.

RECORDING 7

A: What’s your age?

B: 25years

A: Level of education

B: Primary

A: Which Religion?

B: Christianity

A: Are you married

B: No but I have a boyfriend

A: What’s your occupation?

B: Trading

A: Your place of residence

B: Ho Housing

A: Why are you here?

B: I came to a miscarriage. This is the second one

A: Did you have abortion?

B: No, I had a miscarriage

A: How did it happen?

B: the first time, I had an abdominal pain and by the time I got to the hospital, I lost the foetus.

The second one happened after I travelled on a motorbike on an untarred road

A: How much did you spend when you first came to the hospital?

B: I paid GH₵100.00 for the evacuation of the uterus.

A: Who treated you at the hospital?

B: Nurses

A: Were you taken through any counselling?

B: Yes, after the first miscarriage.

A: Have you been taken through Family Planning Services?

B: No

A: Why did you decide to come to this hospital?

B: I Just decided to seek treatment here because they are caring

A: Did you receive any help from anyone and in which form?

B: Yes, my boyfriend provided me with food and took care of all hospital bills.

A: Can you tell me about the attitude of the Health workers?

B: It’s fine. They perform their duties well.

A: Are you satisfied with the services?

B: Yes, because they administer drugs on time and made sure we eat on time. They are always concerned about us.

A: What challenges did you face?

B: unavailability of water at the facility and the far distance from my house to the facility. It will be nice if other health facilities are provided all over town

A: How prompt were you attended to?

B: Very prompt. They came ty aid immediately message got to nurses. I was already in pain so I explained to one of the nurses and she brought here in wheelchair

A: Is the hospital environment clean?

B: Very clean

A: Are there enough beds?

B: Yes

A: Are visitors allowed to visit you?

B: Yes

A: Are you allowed to practice your religion?

B: Yes, people come here to pray for us

A: Any further comments?

B: The water crisis should be resolved so that there can be enough water at the facility

RECORDING 8

A: What’s your age?

B: 30years

A: Level of education

B: Tertiary

A: Which Religion?

B: Christianity

A: Are you married

B: Yes

A: What’s your occupation?

B: Fire officer

A: Your place of residence

B: Ho

A: Have you ever done an abortion?

B: Yes

A: How was the last one done?

B: I went to the hospital. It was just some few weeks so they used the cytotec

A: Was the process easy or you felt pains?

B: Oh you will feel pains no matter what

A: Can you tell me a bit more about it?

B: When you administer the medicine, it takes a longer time for the blood to clot about 9-12hours depending on the stage

A: What’s the cost of the last method and who paid for it?

B: The medicine was around GH₵50.00 and I paid for it.

A: Who delivered the current service to you?

B: The midwife

A: What’s the procedure?

B: It’s normal delivery like when a woman is in labour

A: Did you receive counselling?

B: I received pre-counselling but I was told that they will counsel me again. When I arrived, they told me the membrane around the foetus had damaged so anything could happen; whether good or bad

A: Have you been taken through Family Planning Services?

B: No

A: What made you decide to come for the service?

B: I felt wet so I called my doctor and he asked me to report for an examination

A: Did you receive any help from anyone and in which form?

B: Yes, from my husband and my sister

A: Can you tell me about the attitude of the Health workers?

B: Ever since started coming here, I have never had any problem with them. They are very friendly

A: Are you satisfied with the services?

B: Yes, but the only problem is the water shortage which is a problem in Ho nowadays

A: Did you face any challenges in quest to seek this service?

B: No. I got taxi to bring me here and immediately I arrived, I was given a bed because the doctor was checking on other patients.

He had to skip some patients in order to attend to me before returning to them

A: Are there enough beds?

B: I had bed when I arrived but I don’t know other people’s experiences

A: Are visitors allowed to visit you?

B: Yes, unless one has a peculiar case

A: Are you allowed to practice your religion?

B: Yes, a woman came here to pray for us 2 days ago

A: Why did you choose this facility?

B: This is a referral point. No matter where you go, they will refer you to Trafalgar

A: Any further comments?

B: The sugar is too much for the breakfast

RECORDING 10

A: What’s your age?

B: 33years

A: Level of education

B: Primary

A: Which Religion?

B: Christianity

A: Are you married

B: Yes

A: What’s your occupation?

B: Farming

A: Your place of residence

B: Sokode

A: Have you ever done an abortion?

B: No, I had a miscarriage. The first one happened one happened as a result of a Family Planning method which I stopped because I wanted to give birth again. The current one also happened because of a Family Planning Method which was not effective. I went for the 5years one and I didn’t know it could cause any problem. I had headache and took some paracetamol and later added “sobolo” because my mouth tasted bitter. The following day I felt cold and couldn’t talk so I was rushed to a clinic. I was later transferred to here and I was told I had a miscarriage. They then did the evacuation of the uterus.

A: Who delivered the current service to you?

B: A female doctor

A: What’s the procedure?

B: she injected me, cleaned my vagina before fixing the equipment to evacuate the clot

A: Did you receive counselling?

B: She explained to me that the scan showed there were clots of blood and so must be removed

A: Have you been taken through Family Planning Services?

B: No

A: Did you receive any help from anyone and in which form?

B: Yes, from my husband. He chattered a taxi to convey me to the hospital and he is paying all bills.

A: Can you tell me about the attitude of the Health workers?

B: their attitude is good. They are polite

A: Are you satisfied with the services?

B: Yes, but the only problem is the water shortage which is a problem in Ho nowadays

A: Did you face any challenges in quest to seek this service?

B: water crisis.

A: What can you say about the environment?

B: Very neat

A: Are there enough beds?

B: There are enough beds

A: Are visitors allowed to visit you?

B: Yes, but during visiting hours

A: Are you allowed to practice your religion?

B: Yes. The workers pray with us

A: Any further comments?

B: They should keep up doing the work and replicate it to everyone

RECORDING 11

A: What’s your age?

B: 29years

A: Which Religion?

B: Christianity

A: Are you married

B: Yes

A: What’s your occupation?

B: Seamstress

A: Your place of residence

B: Ho

A: Have you ever done an abortion?

B: No, no abortion yet. I had a quarrel with my husband and the miscarriage happened

A: Who delivered the current service to you?

B: I can’t tell because I was in comma and someone brought me. I later realized I was infused. I felt waist pains

A: Did you receive counselling?

B: the doctor advised me to seek medical attention anytime during pregnancy

A: Have you been taken through Family Planning Services?

B: Yes. They said they have for 5years, 3years and so on

A: When did you decide to seek treatment?

B: I felt waist pain and I saw blood spots in my pants when to urinate. I bled profusely and I became unconscious.

A: Can you tell me about the attitude of the Health workers?

B: Their attitude is good.

A: Are you satisfied with the services?

B: Yes because the treatment was timely

A: What can you say about the environment?

B: I is always cleaned

A: Are there enough beds?

B: There are enough beds

A: Are visitors allowed to visit you?

B: Yes, but during visiting hours

A: Are you allowed to practice your religion?

B: Yes. The workers pray with us

A: why did choose to seek medical attention here?

B: I realized proper care would be given to me here

A: Any further comments?

B: They are doing their best and so they should keep it up.

RECORDING 12

A: What’s your age?

B: 37years

A: Level of Education

B: Informal

A: Which Religion?

B: Christianity

A: Are you married

B: Yes

A: What’s your occupation?

B: Farming

A: Your place of residence

B: Akrofu

A: Have you ever done an abortion?

B: Yes, at the hospital. I can’t talk about the procedure because I was induced with general anaesthesia

A: How much did it cost you and who paid for it?

B: GH₵60.00 and my husband paid for it

A: Did you abort the current pregnancy?

B: No it’s a miscarriage

A: How did it happen?

B: I had an abdominal pain and when I went to toilet, I discovered I was bleeding

A: Who treated you?

B: Doctor through vaginal examination

A: Did you receive counselling?

B: I was advised to take some Family Planning method

A: When did you decide to seek treatment?

B: I get weak when I am pregnant

A: Did you receive help from any source?

B: My husband brought me so he is taking care of everything

A: Can you tell me about the attitude of the Health workers?

B: Their attitude is good.

A: Are you satisfied with the services?

B: Yes. They are calm

A: What can you say about the environment?

B: It is always cleaned

A: Are there enough beds?

B: There are enough beds

A: Are visitors allowed to visit you?

B: Yes, but during visiting hours

A: Did you face any difficulty in getting to the facility?

B: Yes. It was difficult to get car because of the far distance

A: Why did choose to seek medical attention here?

B: I don’t have anybody to stay with me there I decided to come here so that I can receive proper care

A: Any further comments?

B: No

RECORDING 13

A: What’s your age?

B: 29years

A: Level of Education

B: JHS

A: Which Religion?

B: Christianity

A: Are you married

B: Yes

A: What’s your occupation?

B: Trading

A: Your place of residence

B: Dzemeni

A: Have you ever done an abortion?

B: Yes, I have done it once

A: How long did it take to work?

B: It took a short period

A: Was is painful?

B: I felt pains

A: How much did it cost you and who paid for it?

B: GH₵80.00 and my husband paid for it

A: Did you abort the current pregnancy?

B: No it is as result of my BP. It was about six months old.

A: How did it happen?

B: I was given a drug to insert in my vagina

A: Who treated you?

B: Doctor

A: Did you receive counselling?

B: They only talked to me about the BP which was the reason they had to destroy the pregnancy

A: When did you decide to seek treatment?

B: I get weak when I am pregnant

A: Did you receive help from any source?

B: My husband is supporting me financially

A: Can you tell me about the attitude of the Health workers?

B: Their attitude is good.

A: Are you satisfied with the services?

B: Yes. They are calm

A: What can you say about the environment?

B: It is always cleaned

A: Are there enough beds?

B: There are enough beds

A: Are visitors allowed to visit you?

B: Yes, visitors come at 4p.m

A: Did you face any difficulties?

B: No. I was attended to promptly and properly

A: Why did choose to seek medical attention here?

B: I discovered that I became swollen and I was supposed to be operated upon but I refused. I was later referred here. I have come to experience the services. I shall come again anytime I am not feeling well.

A: Any further comments?

B: No

RECORDING 14

A: What’s your age?

B: 28years

A: Level of Education

B: SHS

A: Which Religion?

B: Christianity

A: Are you married

B: No

A: What’s your occupation?

B: Student

A: Your place of residence

B: Tarkwa

A: Have you ever done an abortion?

B: No, it’s a miscarriage

A: Was is painful?

B: I felt pains

A: How much did it cost you?

B: I spent GH₵800.00

A: How did it happen?

B: I felt severe abdominal pain when I returned from the farm. I rushed to the hospital before the foetus had come out. It was six months old

A: Who treated you?

B: Doctor

A: Did you receive counselling?

B:

A: When did you decide to seek treatment?

B: I get weak when I am pregnant

A: Did you receive help from any source?

B: My mother is currently staying with me

A: Can you tell me about the attitude of the Health workers?

B: The workers are wicked. The tell lies and insult people. Sometime ago, they refused to check my BP when it went high until the doctor came

A: Are you satisfied with the services?

B: No, they do not give any proper care

A: What can you say about the environment?

B: The facility is neat

A: Are visitors allowed to visit you?

B: Yes, but they are insulted

A: Did you face any difficulties?

B: It was difficult getting car. The pain started at 12midnight until 2a.m before we got a car to the hospital

A: Why did choose to seek medical attention here?

B: because the hospital isn’t far from where I stay

A: Any further comments?

B: No

RECORDING 15

A: What’s your age?

B: 24years

A: Level of Education

B: JHS

A: Which Religion?

B: Christianity

A: Are you married

B: No

A: What’s your occupation?

B: Seamstress

A: Your place of residence

B: Ho Housing

A: Have you ever done an abortion?

B: I have never done an abortion. I didn’t even know what happened. I am told there is a clot of blood but they haven’t explained it well enough for me. A doctor came and told me there is a clot of blood and so the evacuation of the uterus must be done for me.

RECORDING 16

A: What’s your age?

B: 26years

A: Level of Education

B: SHS

A: Which Religion?

B: Christianity

A: Are you married

B: No

A: What’s your occupation?

B: Fashion Designing

A: Your place of residence

B: Ho

A: Have you ever done an abortion?

B: No but this is the second miscarriage

A: How much did it cost you and who paid for it?

B: GH₵200.00

A: How did it happen?

B: the first I slipped and started bleeding.

A: How were you treated

B: I went to a hospital in Accra and they care of it. They made me laid in bed and some equipment was inserted into my vagina

A: Who treated you?

B: Doctor

A: Did you receive counselling?

B: The doctor was telling me some things when someone interrupted. But he said the foetus had been damaged.

A: What caused the current miscarriage?

B: I can’t tell exactly what happened but I travelled on motorbike and performed some other duties. I later found that I was bleeding. I rushed to the hospital immediately I realized it

A: What happened when you got to the hospital?

B: I was asked to take a scan and was directed to a doctor who advised that it would be safe to terminate the pregnancy because the foetus was damaged.

A: Did you receive any counselling?

B: No

A: Did you receive any Family and Reproductive Health Service?

B: No

A: Why did you go for the service?

B: I knew that it is bad to see blood during pregnancy so I rushed to the hospital immediately

A: Did you receive help from any source?

B: My boyfriend supported me financially

A: Can you tell me about the attitude of the Health workers?

B: Their attitude is okay.

A: Are you satisfied with the services?

B: They were not very prompt anyway but I am satisfied. I will choose Trafalgar again over any other health facility

A: What can you say about the environment?

B: I like the place as compared to other facilities

A: Are there enough beds?

B: I don’t think there are enough beds. I wasn’t admitted. I was came home around 11p.m

A: Are visitors allowed to visit you?

B: Yes

A: Did you face any difficulties?

B: No. I faced no challenges. The distance is normal. No difficulty in getting car to the facility

A: Why did choose to seek medical attention here?

B: I had never been to any government facility in Ho. It was my first and I just knew they might have enough doctors

A: Any further comments?

B: I think should be active nurses in the wards. There were complaints by the senior nurses about the attitude of some junior ones.

RECORDING 17

A: What’s your age?

B: 19years

A: Level of Education

B: JHS

A: Which Religion?

B: Christianity

A: Are you married

B: No

A: What’s your occupation?

B: Student

A: Your place of residence

B: Dave, Ho

A: Have you ever done an abortion?

B: No, this is my first abortion. We were given a dewormer in school according to our heights Our headmaster later informed us that the drug causes abortion.

A: Did you pay for the drug in school?

B: No

A: Who treated you?

B: Doctor

A: Tell me what happened at the first facility

B: I was infused and injected. I was asked to go for a scan. They later referred me to Trafalgar. When I got to Trafalgar, I was told I would be admitted for the evacuation of the uterus. I was given two drugs to swallow instead of a blood transfusion.

A: How was the evacuation done?

B: I was injected on my thigh. A metallic object was inserted through my vagina.

A: Did you receive counselling?

B: I was told not to get pregnant again and was asked to come for a Family Planning method but I didn’t go because I don’t like it

A: When did you decide to seek treatment?

B: Because I had severe abdominal pains

A: Did you receive help from any source?

B: My father helped me financially

A: Can you tell me about the attitude of the Health workers?

B: Their attitude was generally good.

A: Are you satisfied with the services?

B: Yes. They attend to our needs and don’t yell at us

A: What can you say about the environment?

B: It is always cleaned

A: Are there enough beds?

B: There are enough beds

A: Are visitors allowed to visit you?

B: Yes but only when it was time for visitation

A: Did you face any difficulties?

B: Distance was a challenge and the evacuation was not done on time

A: Why did choose to seek medical attention here?

B: I discovered that I became swollen and I was supposed to be operated upon but I refused. I was later referred here. I have come to experience the services. I shall come again anytime I am not feeling well.

A: Any further comments?

B: No

RECORDING 18

A: What’s your age?

B: 24years

A: Level of Education

B: JHS

A: Which Religion?

B: Christianity

A: Are you married

B: No

A: What’s your occupation?

B: Seamstress

A: Your place of residence

B: Housing

A: Have you ever done an abortion?

B: No

A: What brought you to the hospital

B: I was bleeding. I usually don’t have a free flow of blood during my menses

A: Who treated you?

B: Doctor

A: How did he treat you?

B: He checked me up when I came 1 month ago. I had blood transfusion and I was discharged. The problem still existed so I came back on Wednesday. They gave me drugs on Thursday but the clot didn’t come out. I was given another drug to insert in my vagina for it to come. A machine was then plugged and inserted into vagina to extract the clot.

A: Did you receive counselling?

B: No

A: Did you receive any Family Planning Service?

B: No

A: Why did you decide to come to the hospital?

B: Because of the way I was bleeding. I stayed at home for one week before I decided to come to the hospital.

A: Why did you decide to come after one week?

B: I was still bleeding and felt dizzy. I was infused when I arrived

A: Did you receive help from any source?

B: My husband is supporting me financially

A: Can you tell me about the attitude of the Health workers?

B: Some of the nurses are good and some are bad.

A: Are you satisfied with the services?

B: It is satisfactory because the doctor always advises me

A: What can you say about the environment?

B: It is always cleaned

A: Are there enough beds?

B: There are enough beds

A: Are visitors allowed to visit you?

B: Yes, visitors come at 4p.m

A: Did you face any challenges?

B: transportation wasn’t a problem. I wasn’t treated immediately I arrived in the hospital

A: Why did choose to seek medical attention here?

B: I was referred by Ho Polyclinic.

A: Any further comments?

B: Sometimes, there is no sugar in the tea

RECORDING 19

A: What’s your age?

B: 26years

A: Level of Education

B: Tertiary

A: Which Religion?

B: Christianity

A: Are you married

B: single

A: What’s your occupation?

B: Personal Service Person

A: Your place of residence

B: Ho Police Depot

A: Have you ever done an abortion?

B: Yes, I have done it once

A: How did you do it?

B: My boyfriend bought some medicine for me and he asked me to insert it into my vagina

I don’t know the name of the medicine

A: How long did it take to work?

B: I took it around 1p.m and by 8p.m I started facing complication

A: How much did it cost you and who paid for it?

B: No, I don’t he (my boyfriend) paid for it

A: Who delivered the current post abortion care service to you?

B: A doctor

A: What exactly brought you to the hospital?

B: After the abortion, I begun to bleed and experience severe abdominal pain. So when the doctor checked, he said it was an incomplete abortion and I had to be taken through series of treatment.

A: How was the treatment done for you?

B: some metallic objects were passed through my private part and brought out some things

A: Was the process painful?

B: It was very painful. in fact, I couldn’t bear it. I just felt like dying.

A: Did you receive counselling?

B: Yes, I was taken the Family Planning Department and a doctor advised me on how to protect premarital pregnancies. He said they have pills that I could take, condoms and the injectable for 3months, 6months upwards to prevent unwanted pregnancies.

A: What made you come for the service?

B: I felt I needed advice on how to manage my sexual activities because I didn’t want the same situation to happen again

A: Did you receive help from any source?

B: My dad assisted. He supported me and took care of me

A: Can you tell me about the attitude of the Health workers?

B: They were very nice. I thought they would get angry and yell at me but thyey really made me feel comfortable.

A: Are you satisfied with the services?

B: Yes. They really took care of me and gave me the necessary advice I needed. I also received early treatment

A: What can you say about the environment?

B: It is a clean place

A: Are there enough beds?

B: There are enough beds

A: Are visitors allowed to visit you?

B: Yes, visitors come at 4p.m

A: Did you face any difficulties?

B: I didn’t really notice that but I think there was difficulty in finding a car to the facility

A: Why did choose to seek medical attention here?

B: It is the biggest hospital in the Volta Region. I would prefer to be in Trafalgar because I would be assured of the proper care needed

A: Any further comments?

B: No

RECORDING 20

A: What’s your age?

B: 26years

A: Level of Education

B: Tertiary

A: Which Religion?

B: Christianity

A: Are you married

B: In a relationship

A: What’s your occupation?

B: Teaching

A: Your place of residence

B: Medical Village, Ho

A: Have you ever done an abortion?

B: Yes

A: How did you do it?

B: I swallowed 2 pills and inserted 2 into my vagina

A: Do you know the name of the pill?

B: No. I don’t

A: What happened after taking the pill?

B: After about 45minutes, I started bleeding

A: How long did it take to work?

B: It took a short period

A: Was the method easy?

B: It was quite easy but after some hours, it was painful

A: How much did it cost you and who paid for it?

B: The causer (my boyfriend) paid for it. I think it cost him over a GH₵100.00

A: What made you come to the hospital?

B: I was bleeding excessively so I knew there was something wrong so I came to the hospital

A: Who treated you?

B: Doctor

A: What treatment was given to you?

B: He inserted something into my vagina because he said it was an incomplete abortion

A: Did you receive counselling?

B: Yes. They educated me about some of the pregnancy prevention methods

The use of pills, condoms

A: When did you decide to seek treatment?

B: I get weak when I am pregnant

A: Did you receive support from anybody in seeking post abortion care?

B: My boyfriend brought me to the hospital and supported me by paying all bills. He showed concern

A: Can you tell me about the attitude of the Health workers?

B: They have a good attitude. Their response was so fast.

A: Are you satisfied with the services?

B: Yes. The treatment was very good

A: What can you say about the environment?

B: It is always cleaned

A: Are there enough beds?

B: There are enough beds

A: Are visitors allowed to visit you?

B: Yes, visitors come at 4p.m

A: Did you face any difficulties?

B: my house isn’t far from the hospital so I didn’t have any challenge with transportation. I wouldn’t say I was treated fast but it was normal. They actually took their time

A: Are religious activities allowed in the facility?

B: I don’t know because I left that very day

A: Why did you choose to seek medical attention here?

B: Because the hospital is close to house and it’s the Regional Hospital.

RECORDING 21

A: What’s your age?

B: 26years

A: Level of Education

B: Tertiary

A: Which Religion?

B: Christianity

A: Are you married

B: In a relationship

A: What’s your occupation?

B: Estate Officer

A: Your place of residence

B: Sokode Lokoe

A: Have you ever done an abortion?

B: Yes, once

A: How did you do it?

B: I inserted a drug and the foetus came out after 3 hours. I started bleed

A: Was the method easy?

B: It was quite easy. A little blood came out at the beginning and there was complication.

A: How much did it cost you and who paid for it?

B: My boyfriend paid for it. It was about GH₵200.00

A: What made you come to the hospital?

B: I did not decide to come My boyfriend brought me

A: Who treated you?

B: Doctor

A: What treatment was given to you?

B: I was given a pill to swallow and I laid down for about 25minutes and I began to bleed

A: Did you receive counselling?

B: Yes. They educated me about how to abstain or use pills or condoms to avoid pregnancy

A: Did you receive support from anybody in seeking post abortion care?

B: My boyfriend because I was trying to hide it from my parents. He paid for all the services

A: Can you tell me about the attitude of the Health workers?

B: They were not friendly since they knew that you were trying abortion, they wouldn’t treat you well.

A: Are you satisfied with the services?

B: No. The nurses were not caring and they were harsh

A: Did you face any difficulties?

B: It was a difficult task getting a taxi to the hospital. I waited for about 30 minutes before I was attended to

A: Why did you choose to seek medical attention here?

B: I came because it was an emergency. I was unconscious so I was brought here.

RECORDING 22

A: What’s your age?

B: 25years

A: Level of Education

B: Tertiary

A: Which Religion?

B: Christianity

A: Are you married

B: Married

A: What’s your occupation?

B: Trader

A: Your place of residence

B: Lome

A: Have you ever done an abortion?

B: No. This is miscarriage, I dreamt that that I was attacked by some creature and when I woke up, I was bleeding. We visited the hospital and I was injected. I took a scan and I was told I had lost the pregnancy

A: Was it difficult?

B: It wasn’t difficult except yesterday when I began feeling severe abdominal pains

A: How much did it cost you and who paid for it?

B: I spent up to 1000.00CFA

A: What made you come to the hospital?

B: I was bleeding excessively so I knew there was something wrong so I came to the hospital

A: Who treated you?

B: Doctor

A: What treatment was given to you?

B: Something was used to remove the clot but I didn’t see it because I was induced

A: Did you receive counselling?

B: They only told I would feel a little pain

A: Did you receive any Family Planning Services

B: No

A: Did you receive support from anybody in seeking post abortion care?

B: My mother is with me. My aunt brought some money to pay the bills

A: Can you tell me about the attitude of the Health workers?

B: They have good attitude.

A: Are you satisfied with the services?

B: I am satisfied because I have received help. I am only afraid I would be able to give birth

A: Did you face any difficulties?

B: No difficulty is transportation and response was quick

A: Why did you choose to seek medical attention here?

B: My husband asked me to come here. I didn’t know this facility because I come from Togo but husband comes from Agortime so he recommended this place to us.

RECORDING 23

A: What’s your age?

B: 27years

A: Level of Education

B: JHS

A: Which Religion?

B: Christianity

A: Are you married

B: Single

A: What’s your occupation?

B: Unemployed

A: Your place of residence

B: Tsereboni

A: Have you ever done an abortion?

B: No. I have never done an abortion except this one. The membrane surrounding the foetus got damaged so I went to the hospital and the health workers destroyed the pregnancy. It was five months.

A: Who treated you?

B: Doctor

A: What treatment was given to you?

B: I was given a drug to swallow and also insert in my private part. I bled afterwards. I was then given another drug to help stop the blood. I took the drug at 6p.m and by 3:30a.m, everything was passed out

A: Did you receive counselling or Family Planning Service?

B: I was only told to pass by the centre when I had been discharged and going home

A: Did you receive support from anybody in seeking post abortion care?

B: My sister brought me to this place and she has been with me since then. My boyfriend is paying all bills

A: What can you say about the attitude of the workers?

B: Their attitude is good. They are caring and do not chastise me

A: Can you tell me about the attitude of the Health workers?

B: They have good attitude.

A: Are you satisfied with the services?

B: Yes because of the prompt response

A: Did you face any difficulties?

B: I didn’t face any challenge in terms of transportation

A: What can you say about the environment?

B: It is neat. The wards and washrooms are always cleaned

A: Are visitors allowed to visit?

B: only during visiting hours

A: Are you allowed to practice your religion?

B: People are allowed to come and pray with us. A Pastor came yesterday.

A: Why did you choose to seek medical attention here?

B: We went to a clinic in my area and they referred us to Trafalgar
